# Oxidative radical ring-opening/cyclization of cyclopropane derivatives

**DOI:** 10.3762/bjoc.15.23

**Published:** 2019-01-28

**Authors:** Yu Liu, Qiao-Lin Wang, Zan Chen, Cong-Shan Zhou, Bi-Quan Xiong, Pan-Liang Zhang, Chang-An Yang, Quan Zhou

**Affiliations:** 1 Department of Chemistry and Chemical Engineering, Hunan Institute of Science and Technology, Yueyang 414006, P. R. China

**Keywords:** cyclopropane derivatives, free radicals, ring-opening/cyclization

## Abstract

The ring-opening/cyclization of cyclopropane derivatives has drawn great attention in the past several decades. In this review, recent efforts in the development of oxidative radical ring-opening/cyclization of cyclopropane derivatives, including methylenecyclopropanes, cyclopropyl olefins and cyclopropanols, are described. We hope this review will be of sufficient interest for the scientific community to further advance the application of oxidative radical strategies in the ring-opening/cyclization of cyclopropane derivatives.

## Introduction

**Scheme 1:**
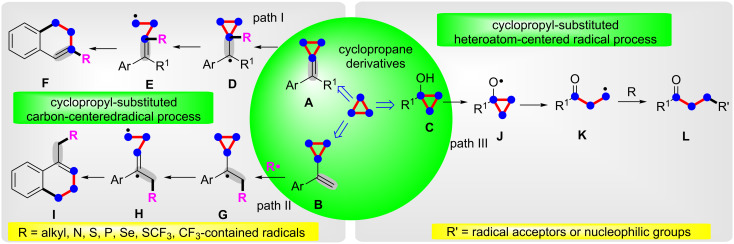
The oxidative radical ring-opening/cyclization of cyclopropane derivatives.

Cyclopropane is a cycloalkane molecule with the molecular formula C_3_H_6_, consisting of three carbon atoms linked to each other to form a ring, with each carbon atom bearing two hydrogen atoms resulting in *D*_3_*_h_* molecular symmetry. The small size of the ring creates substantial ring strain in the structure. The cyclopropane skeleton easily can take part in ring-opening reactions under certain conditions. Cyclopropane derivatives, with their three-membered carbocyclic frameworks, have spurred considerable attention especially in the domain of organic and pharmaceutical synthesis because of their highly strained three-membered carbocyclic skeletons and their easy availability [1–16]. The cyclopropane derivatives, especially methylenecyclopropanes [17–21], cyclopropyl olefins [22] and cyclopropanols [23–24] undergo ring-opening/cyclization reactions to provide a huge number of fascinating compounds with different functional groups [25–31]. However, most recently reported methods usually proceed via a radical pathway. As shown in Scheme 1 path I, the cyclopropyl-substituted carbon radical **D** is formed by the addition of radical **R** to the C–C double bond in methylenecyclopropanes (compounds **A**). The cyclopropyl-substituted carbon radical **D** easily goes through a ring-opening to generate the alkyl radical **E**, and then cyclizes with the phenyl ring to afford the terminal product **F** (path I). The cyclopropyl olefins (compounds **B**) also react in the same cyclopropyl-substituted carbon radical pathway to finish the ring-opening and cyclization transformation (path II). The cyclopropanols **D** firstly go through homolytic cleavage of the O–H bond to give the oxygen-centered radical **J**. The alkyl radical **K**, produced by ring-opening of intermediate **J**, reacts with a radical acceptor or a nucleophilic group to obtain the product **L** (path III).

Free radical reactions have flourished and became a powerful tool in organic synthesis [32–38]. With the significant potential, this strategy has captured the human’s attention and solved considerable problems in the past several decades [39–42]. The free radical reaction was applied in a range of organic transformations because of its unique advantages such as excellent reactivity, mild conditions, functional group tolerance, and atom economy. A series of radicals, such as carbon, Se, CF_3_, halogen, S and N-containing radicals, were introduced into the products through oxidative radical ring-opening/cyclization of cyclopropane derivatives. In this review, we conclude recent advance in the oxidative radical ring-opening/cyclization of cyclopropane derivatives (including methylenecyclopropanes, cyclopropyl olefins and cyclopropanols) over the last 20 years.

## Review

### Oxidative radical ring-opening and cyclization of methylenecyclopropanes (MCPs)

**Scheme 2:**
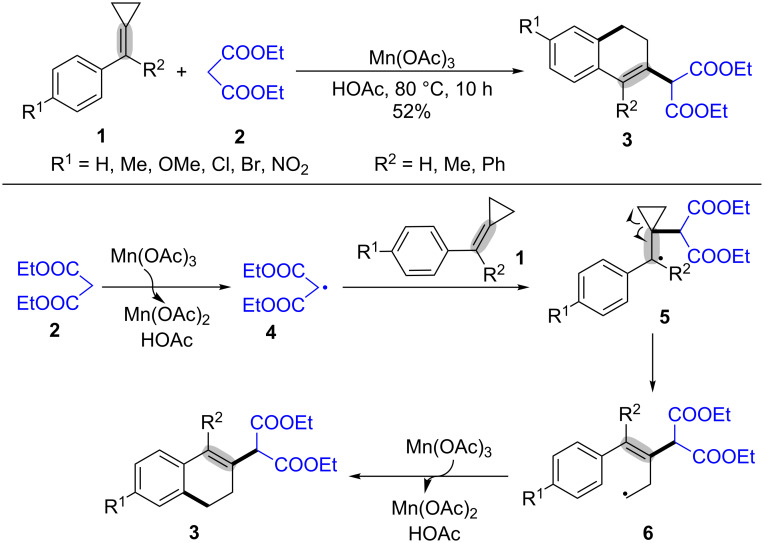
Mn(OAc)_3_-mediated oxidative radical ring-opening and cyclization of MCPs with malonates.

In 2004, Huang and co-workers reported the first manganese(III) acetate-mediated radical ring-opening and cyclization of methylenecyclopropanes (MCPs, **1**) with malonic acid diethyl esters (**2**, Scheme 2) [43]. This strategy provided a novel, convenient and efficient approach to construct 2-(3,4-dihydronaphthalen-2-yl)malonic acid diethyl esters **3**. The MCPs **1** with the electron-deficient or electron-rich groups were all suitable for this reaction system. The mechanism for the Mn(OAc)_3_-mediated oxidative radical ring-opening and cyclization of MCPs with malonates is outlined in Scheme 2. Initially, the malonic acid diethyl ester (**2**) was transformed into radical **4** [44] under the action of Mn(OAc)_3_. Then, the selective addition of the radical **4** to the C–C double bond of MCPs **1** formed the more stable benzyl radical intermediate **5** [45–46], which underwent a ring-opening to generate the alkyl radical **6** [47]. Finally, the desired product **3** was generated through intramolecular cyclization of radical intermediate **6** with an aryl ring and oxidation deprotonation by another molecule Mn(OAc)_3_ [48].

**Scheme 3:**
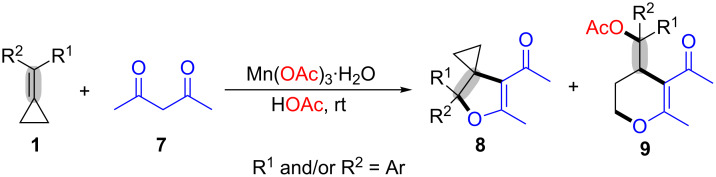
Mn(III)-mediated oxidative radical ring-opening and cyclization of MCPs with 1,3-dicarbonyl compounds.

Later, Shi et al. demonstrated an oxidative annulation of MCPs **1** with 1,3-dicarbonyl compounds **7** using manganese(III) catalysis under room temperature conditions, which afforded 4,5-dihydrofuran derivatives **8** as [3 + 2] annulation products (cyclopropyl retained adducts) in moderate to good yields [49]. This transformation also gave another six-membered cyclic compounds **9** (cyclopropyl opened adducts) via ring-opening and cyclization process (Scheme 3). However, the [3 + 2] annulation reaction did not occur under the standard conditions when the MCPs **1** was without an aromatic group.

**Scheme 4:**
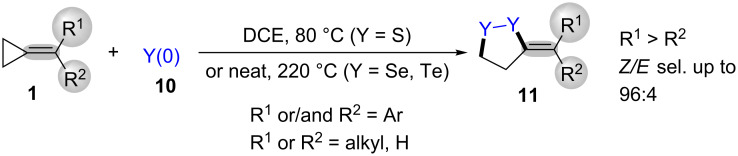
Heat-promoted ring-opening/cyclization of MCPs with elemental chalgogens.

The first method for direct [3 + 2] radical cycloaddition of MCPs **1** with elemental chalcogens **10** (S, Se, Te) was developed by Yu and co-workers. This strategy presented a simple and efficient method for the synthesis of methylene-1,2-dichalcogenolanes **11** (Scheme 4) [50]. This reaction proceeded via a radical pathway, which could take place smoothly under cartalyst- and additive-free conditions. However, the addition of the radical initiator AIBN in this reaction did not accelerate the reaction.

**Scheme 5:**
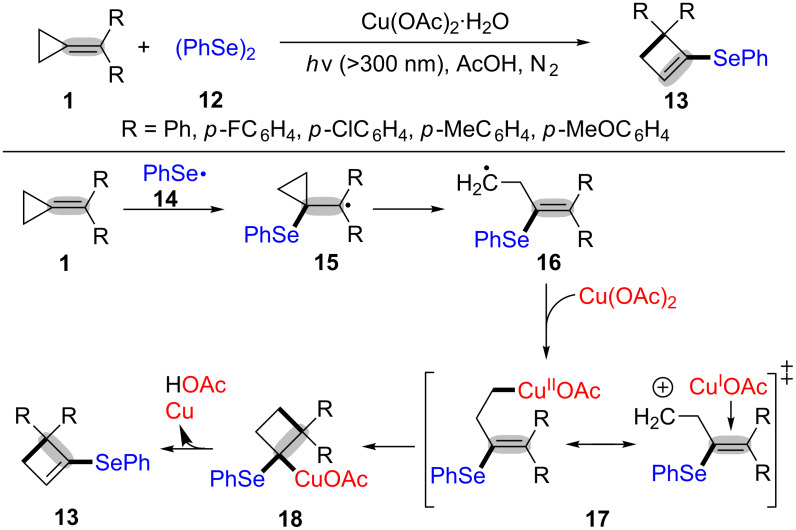
Copper(II) acetate-mediated oxidative radical ring-opening and cyclization of MCPs with diphenyl diselenides.

Next, Huang’s group proposed the copper-catalyzed ring-opening and cyclization of MCPs **1** with diphenyl diselenides **12** for the synthesis of 2-phenylseleny-3,3-diarylcyclobutenes **13** under visible light irradiation (Scheme 5) [51]. The desired products **13** contained a cyclobutene group and a selenium atom, which makes the products possess unique biological and pharmaceutical activities. The mechanism of the copper(II) acetate-mediated oxidative radical ring-opening/cyclization of MCPs with diphenyl diselenides is outlined in Scheme 5. Firstly, the phenylselenyl radical **14**, generated from the homolytic cleavage of diphenyl diselenide, is added to the C–C double bond of MCPs to afford the intermediate **15**, which undergoes a ring-opening process to form the radical intermediate **16** [52–53]. Then, the radical **16** reacts with copper(II) acetate to produce organocopper intermediate **17**. Finally, the intramolecular insertion of C–Cu in compounds **17** to the carbon–carbon double bond takes place to produce the intermediate **18** followed by β-elimination to generate the desired product **13** [54–56].

**Scheme 6:**
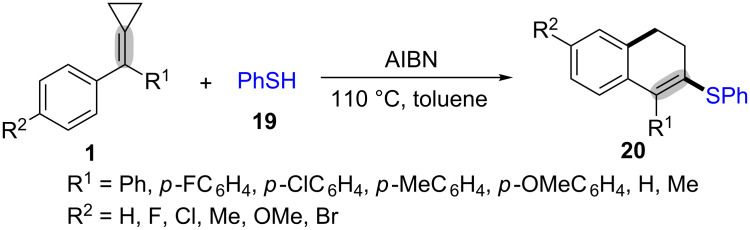
AIBN-promoted oxidative radical ring-opening and cyclization of MCPs with benzenethiol.

In 2005, Yu et al. described a novel and efficient oxidative radical ring-opening and cyclization of MCPs **1** with benzenethiol (**19**) for the synthesis of 3-phenylsulfanyl-1,2-dihydronaphthalenes **20** in moderate to good yields (Scheme 6) [57]. Additionally, using benzeneselenol instead of benzenethiol under the standard conditions generated the corresponding products in 31% yields.

**Scheme 7:**
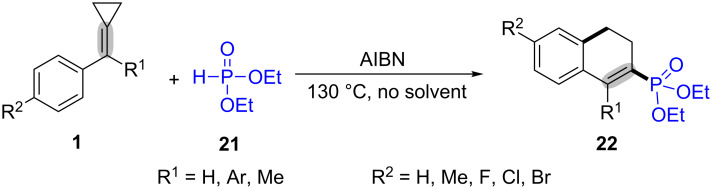
AIBN-mediated oxidative radical ring-opening and cyclization of MCPs with diethyl phosphites.

In the same year, Huang’s group also reported a similar ring-opening and cyclization of MCPs **1** with diethyl phosphites **21** for building diethyl 3,4-dihydro-2-naphthylphosphonates **22** (Scheme 7) [58]. This was the first example to synthesize the diethyl 3,4-dihydro-2-naphthylphosphonates **22** that have great potential applications in organic chemistry and biochemistry.

**Scheme 8:**
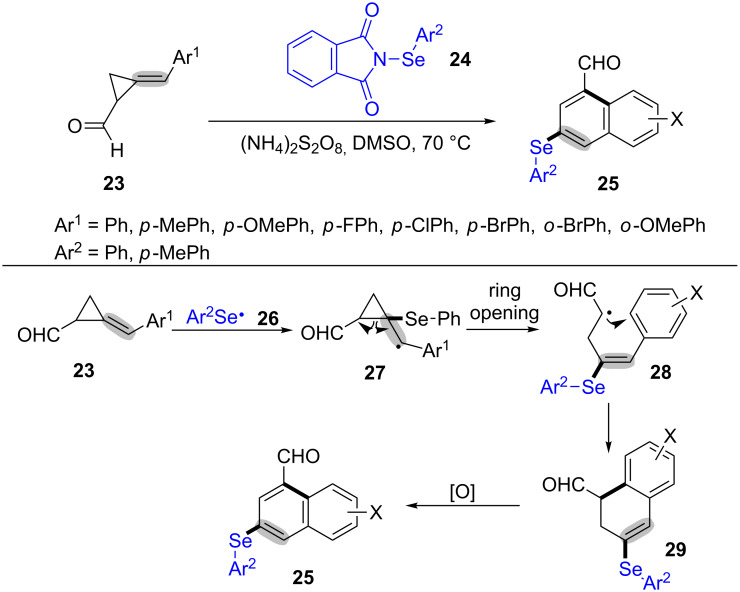
Organic-selenium induced radical ring-opening and cyclization of MCPs derivatives (cyclopropylaldehydes).

In 2009, Miao’s group also discovered another method for the synthesis of 1-naphthaldehydes **25** under mild conditions via a radical-mediated ring-opening and intramolecular cyclization of MCPs **23** with organic selenium reagents **24** (Scheme 8) [59]. In this reaction, the MCPs with electron-withdrawing groups gave lower yields than that with electron-donating groups. Additionally, the use of other organoselenium reagents, such as phenylselenyl bromide or phenylselenyl chloride provided only trace amounts of the desired products. The mechanism for the organoselenium induced radical ring-opening and cyclization of MCPs derivatives is showed in Scheme 8. Firstly, phenylselenyl radical **26** was produced in the presence of free radical initiator (NH_4_)_2_S_2_O_8_ [60–61]. Next, the intermediate **26** was added to the C–C double bond of MCPs **23**, and then went through a series of ring-opening, intramolecular cyclization, oxidation and dehydrogenation to generate 3-arylselanylnaphthaldehyde **25**.

**Scheme 9:**
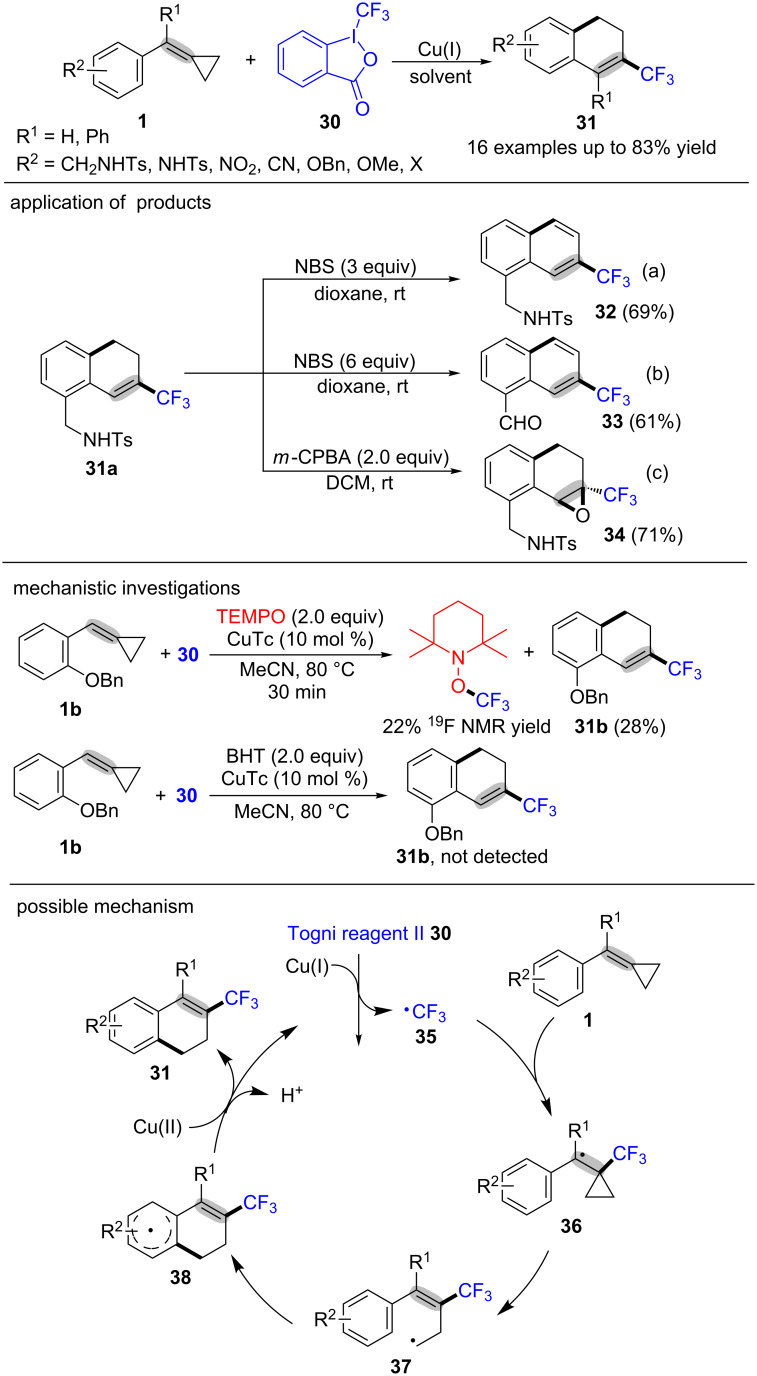
Copper(I)-catalyzed oxidative radical trifluoromethylation/ring-opening/cyclization of MCPs with Togni reagent II.

In 2015, Shi and co-workers reported a novel and efficient method to construct CF_3_-substituted dihydronaphthalene derivatives **31** in moderate to excellent yields under mild conditions through the Cu(I)-catalyzed trifluoromethylation/ring-opening/cyclization of MCPs **1** with Togni reagent II (**30**, Scheme 9) [62]. In this transformation, many substituted MCPs **1** with alkyl groups, Ts-protected amino groups, or halogens were tolerated well and gave the desired products **31** in good yields. Moreover, the product **31a** could go through a further oxidation to afford two different products in the presence of different amount of NBS (*N*-bromosuccinimide). The corresponding CF_3_-substituted naphthalene **32** could be obtained in 69% yield when the product **31a** was oxidized by 3 equiv of NBS (Scheme 9, reaction a). When the amount of NBS was increased to 6 equiv under identical conditions, the CF_3_-substituted naphthaldehyde **33** was obtained in 61% yield (Scheme 9, reaction b). Furthermore, the product **31a** could also be transformed to the CF_3_-substituted epoxide **34** in the presence of 2 equiv *m*-CPBA (*m*-chloroperbenzoic acid) (Scheme 9, reaction c). A radical-trapping experiment was conducted with the addition of TEMPO or BHT under the standard conditions, and the reactions were suppressed by radical scavengers, which suggested that the reaction underwent a radical process. The proposed mechanism is depicted in Scheme 9. Initially, the CF_3_ radical **35** is generated from the Togni reagent II (**30**) under the action of Cu(I) [63–64]. Then the CF_3_ radical **35** adds to the C–C double bond in MCPs **1** to give the more stable benzyl radical intermediate **36** which went through a ring-opening process to provide the alkyl radical intermediate **37**. The intermediate **37** undergoes intramolecular cyclization with the aromatic ring to generate intermediate **38** which is oxidized by Cu(II) to provide the CF_3_-substituted dihydronaphthalenes derivatives **31** along with releasing a proton [65–66].

**Scheme 10:**
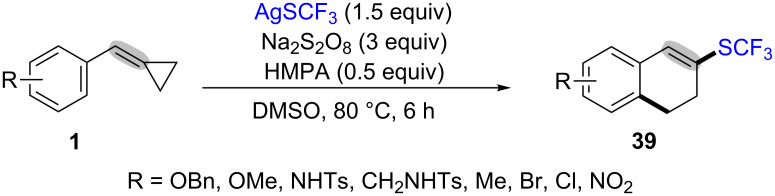
Ag(I)-mediated trifluoromethylthiolation/ring-opening/cyclization of MCPs with AgSCF_3_.

The trifluoromethylthiolation of MCPs **1** with AgSCF_3_ was achieved by Shi et al. which proceeds through a sequence of radical addition, ring-opening, cyclization, oxidation and dehydrogenation and successfully furnished trifluoromethylthiolated 1,2-dihydronaphthalene derivatives **39** (Scheme 10) [67]. This reaction was achieved in the presence of 3.0 equiv of Na_2_S_2_O_8_ as the oxidants, 0.5 equiv of HMPA (*N*,*N*,*N*',*N*',*N*'',*N*''-hexamethylphosphorotriamide) as the additive in DMSO.

**Scheme 11:**
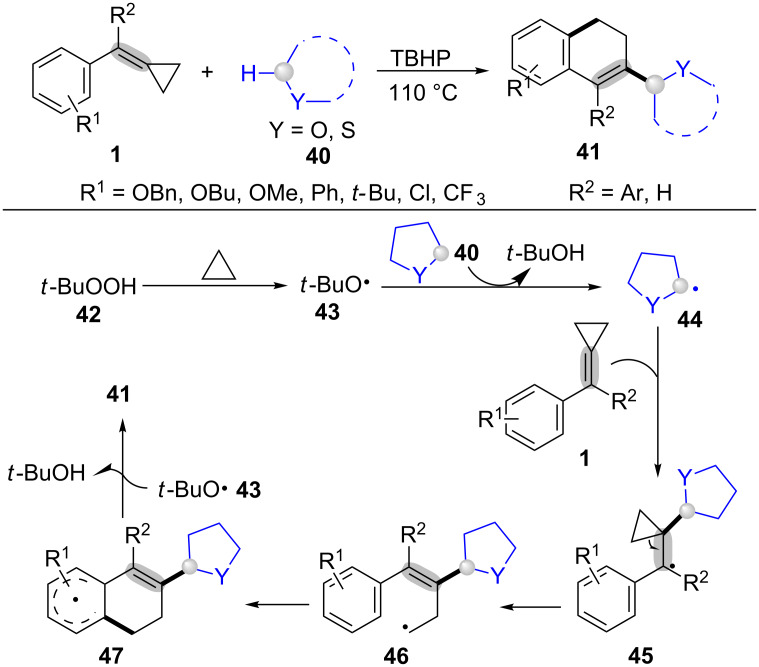
oxidative radical ring-opening and cyclization of MCPs with α-C(sp^3^)-–H of ethers.

With a similar oxidative radical ring-opening and cyclization strategy, our group developed a novel method for ring-opening and cyclization of MCPs **1** with ethers **40** afforded 2-substituted 3,4-dihydronaphthalenes **41** in moderate to excellent yields (Scheme 11) [68]. This transformation just needed 2 equiv of TBHP (**42**), avoiding using transition metal catalysts, ligands, and bases. In the proposed mechanism (Scheme 11), the *tert*-butoxyl radical **43**, which was formed from THBP (**42**) under heating conditions, attackes the ether **40** to afford the radical **44** [69–72]. Next, the addition of radical **44** to the C–C double bond of MCPs **1** generats a more stable benzyl radical **45**. Final ring-opening, intramolecular cyclization, oxidation, and dehydrogenation finally delivers the desired product **41**.

**Scheme 12:**
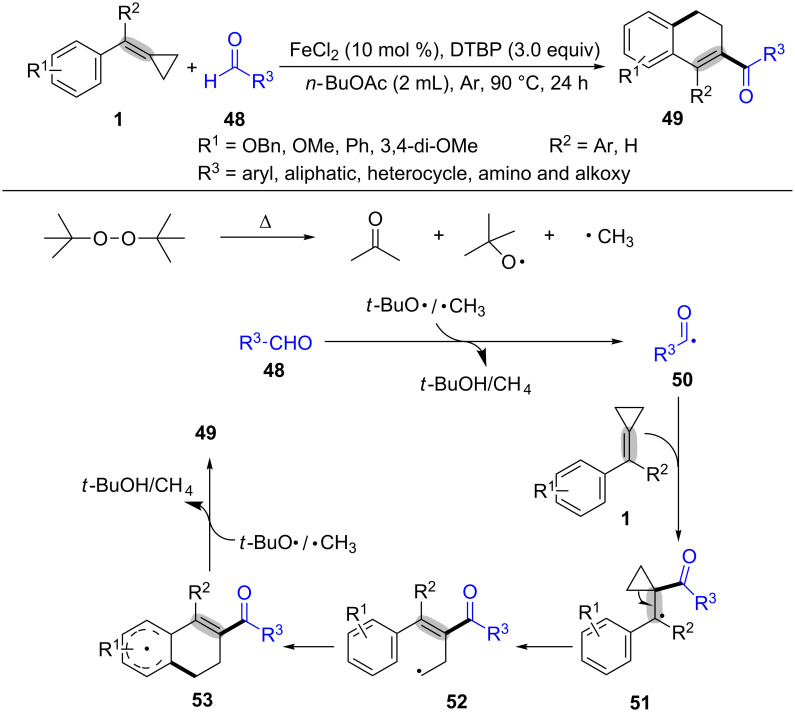
Oxidative radical ring-opening and cyclization of MCPs with aldehydes.

Next, our group reported the first oxidative ring-opening and cyclization between MCPs **1** and aldehydes **48** to provide 2-acyl-3,4-dihydronaphthalenes **49** in moderate to excellent yields via a series of radical addition, ring-opening and cyclization in the presence of DTBP (di-*tert*-butyl peroxide) and Lewis acids (Scheme 12) [73]. Moreover, the experimental results showed MCPs **1** with electron-rich aryl groups could deliver higher yields than that with electron-deficient ones. As outlined in Scheme 12, a *tert*-butoxy radical and a methyl radical were generated from cleavage of DTBP at the reaction temperature. Aldehyde **48** is easily transformed into acyl radical **50** in the presence of an alkoxy radical or a methyl radical [74–77]. The acyl radical **50** adds to the C–C double bond of MCPs giving the benzyl radical intermediate **51**. The ring-opening of radical intermediate **51** occurres to form the alkyl radical intermediate **52** which intermolecularly cyclizes with the aryl ring. The following oxidation and dehydrogenation gives the target product **49**.

**Scheme 13:**
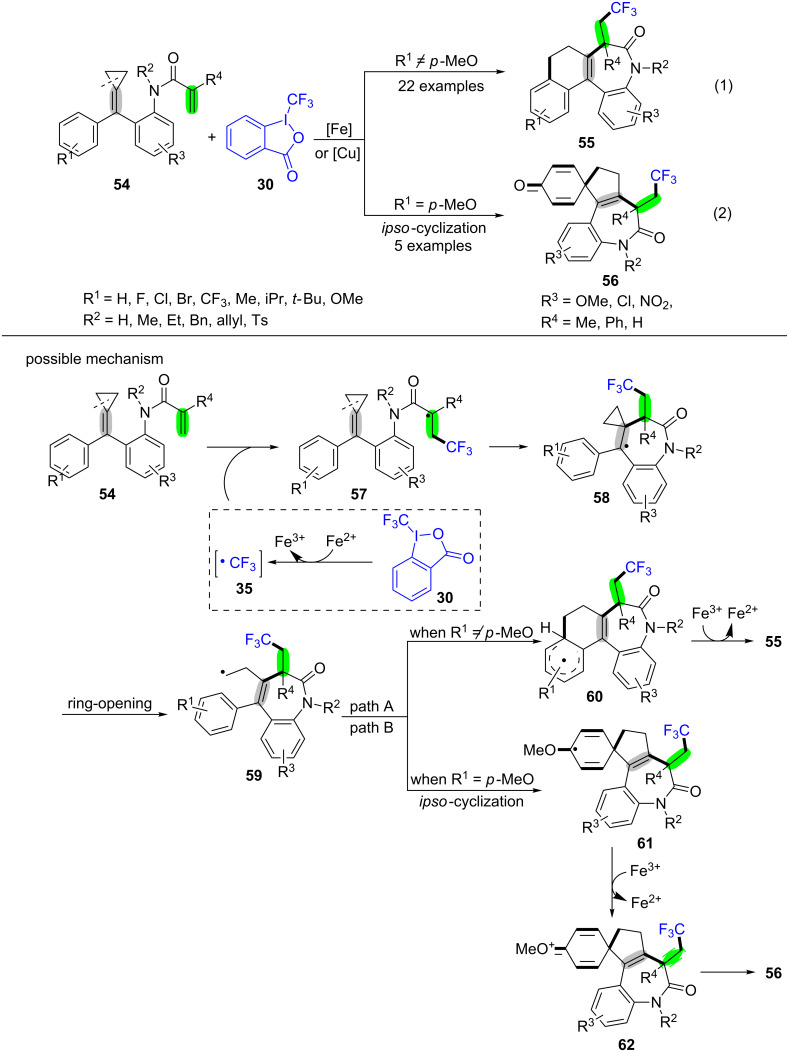
Cu(I) or Fe(II)-catalyzed oxidative radical trifluoromethylation/ring-opening/cyclization of MCPs derivatives (acrylamide-tethered alkylidenecyclopropanes).

A new and first achievement for the synthesis of CF_3_-contained seven-membered ring compounds **55** and **56** through trifluoromethylation of acrylamide-tethered alkylidenecyclopropanes **54** was presented by Shi and co-workers (Scheme 13) [78]. The possible reaction pathway is outlined in Scheme 13. Initially, the Togni reagent II (**30**) goes through a single-electron transfer (SET) under the action of Fe^2+^ to generate the CF_3_ radical **35**. The CF_3_ radical **35** is trapped by the C–C double bond of substrate **54** to produce the alkyl radical intermediate **57**. Then, the intramolecular addition of an alkyl radical to the less hindered central carbon of MCPs **54** gives the benzyl radical intermediate **58**, which undergoes a ring-opening process to provide the alkyl radical intermediate **59** [79–80]. Because of the different substituent groups on the MCPs **54** (whether R^1^ was a *para*-methoxy substituent or not), this reaction proceeds through two different pathways. When R^1^ is not a *para*-methoxy group, the intermediate **59** undergoes a conventional cyclization with aromatic ring to afford the radical intermediate **60**. After oxidation and aromatization, the corresponding product **55** is formed. An *ipso*-cyclization with aromatic ring occurres and gives the intermediate **61** when R^1^ is a *para*-methoxy group. The oxonium ion **62** is produced by the oxidation of the intermediate **61** under the action of Fe^3+^ [81]. Lastly, the oxonium ion **62** is transformed into the desired product **56** in the presence of 2-indobenzoic acid anion.

**Scheme 14:**
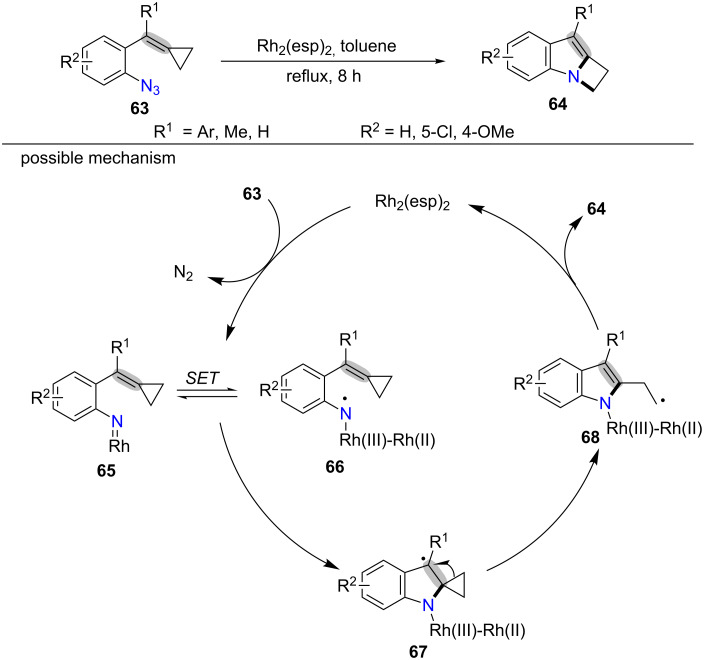
Rh(II)-catalyzed oxidative radical ring-opening and cyclization of MCPs.

Recently, Shi’s group developed the first ring expansion of MCPs **63** with a nitrogen atom to furnish azetidines **64** (Scheme 14) [82]. The author proposed that Rh(II) had an effective impact on the reactions and could improve the reaction yields. Unfortunately, the MCPs **63** with the groups R^1^ and R^2^ = H were not suitable for this transformation. The reason was because the formed intermediate was unstable under this conditions. A possible mechanism is outlined in Scheme 14. Initially, the Rh-nitrene intermediate **65** [83–86] is generated from the coordination of azide to Rh_2_(esp)_2_ complex (bis[rhodium-(α,α,α’,α’-tetramethyl-1,3-benzenedipropionic acid)]) and extrusion of N_2_. Then, the Rh-nitrene intermediate **65** goes through an intramolecular single electron transfer (SET) to give the nitrogen-centered radical intermediate **66** [87–90]. Next, the radical addition of intermediate **66** to the C–C double bond in MCPs moiety furnishes the more stable benzyl radical intermediate **67**, which is ring-opened to give alkyl radical **68**. Finally, intermediate **68** goes through SET with the Rh(III) species and intramolecular cyclization with the 2-position of the indole moiety to afford the target product **64** along with the regenerated Rh(III) catalyst.

**Scheme 15:**
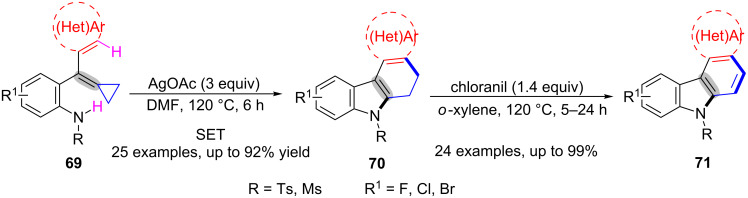
Ag(I)-catalyzed oxidative radical amination/ring-opening/cyclization of MCPs derivatives.

A silver-catalyzed intramolecular cascade amination/ring-opening/cyclization of a variety of substituted MCPs **69** was proposed by Fan and co-workers, which provided a simple and efficient way for the building of [2,3-*c*]dihydrocarbazoles **70** and [2,3-*c*]carbazoles **71** (Scheme 15) [91]. This process permitted the use of readily available and cheap AgOAc as the catalyst and oxidant, and DMF as the solvent. Notably, the product **70** was easily transformed into **71** in the presence of chloranil (1.4 equiv) at 120 °C under Ar atmosphere for 5 h. In this transformation, substrates with electron-donating groups showed higher yields than the ones with electron-withdrawing groups.

**Scheme 16:**
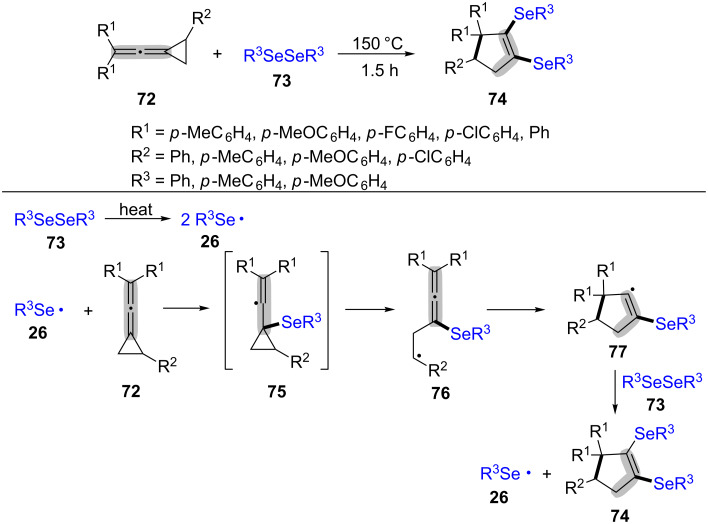
Heating-promoted radical ring-opening and cyclization of MCP derivatives (arylvinylidenecyclopropanes) with diaryl diselenides.

In the same year, Shi et al. reported an effective ring-opening and cyclization of arylvinylidenecyclopropanes **72** with diaryl diselenides **73** for the synthesis of 1,2-diarylselenocyclopentene **74** in moderate to good yields at 150 °C for 1.5 h (Scheme 16) [92]. The electron-rich, electron-neutral and electron-poor arylvinylidenecyclopropanes were tolerated well in this transformation. The detailed mechanism is outlined in Scheme 16. Initially, the homolysis of diphenyldiselenide **73** under heating conditions produces the phenylseleno radical **26** [93]. Then, the additon of radical **26** to the C–C double bond of MCPs derivatives **72** affords the radical intermediate **75** [94]. Next, the radical **75** goes through a ring-opening process to give the radical intermediate **76**. The intermediate **77**, produced by the intramolecular cyclization of intermediate **76**, reactes with diphenyl diselenide **73** to form the target product **74** via homolytic substitution (S_H_).

**Scheme 17:**
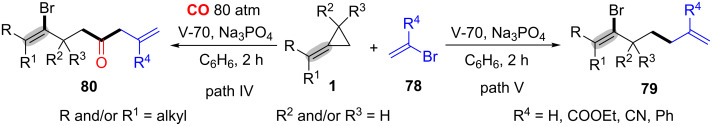
Bromine radical-mediated ring-opening of alkylidenecyclopropanes.

In 2013, Ryu and co-workers developed the bromine radical-mediated ring-opening and alkylation of alkylidenecyclopropanes **1** with allylic bromides **78** for the synthesis of 2-bromo-1,6-dienes **79** via radical ring-opening and S_H_2’ reactions (path V in Scheme 17) [95]. The experimental results suggested that radical carbonylation could also be incorporated in the reaction sequence, leading to 2-bromo-1,7-dien-5-ones **80** (path IV in Scheme 17).

**Scheme 18:**
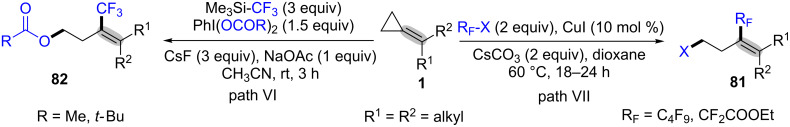
Fluoroalkyl (Rf) radical-mediated ring-opening of MCPs.

In 2016, Xu’s group exploited the fluoroalkyl (R_F_) radical-mediated ring-opening of MCPs **1** for the synthesis of fluorinated homoallylic compounds (**80** and **81**, Scheme 18) [96]. In this reaction system, the radical reaction of MCPs **1** with R_F_-X (X = Br, I) furnished homoallylic halides in excellent yields (path VII in Scheme 18). Similarly, the radical reaction of MCPs **1** with the R_F_TMS/CsF/PhI(OAc)_2_ gave homoallylic acohol esters in moderate to good yields (path VI in Scheme 18).

### Oxidative radical ring-opening and cyclization of cyclopropyl olefins

**Scheme 19:**
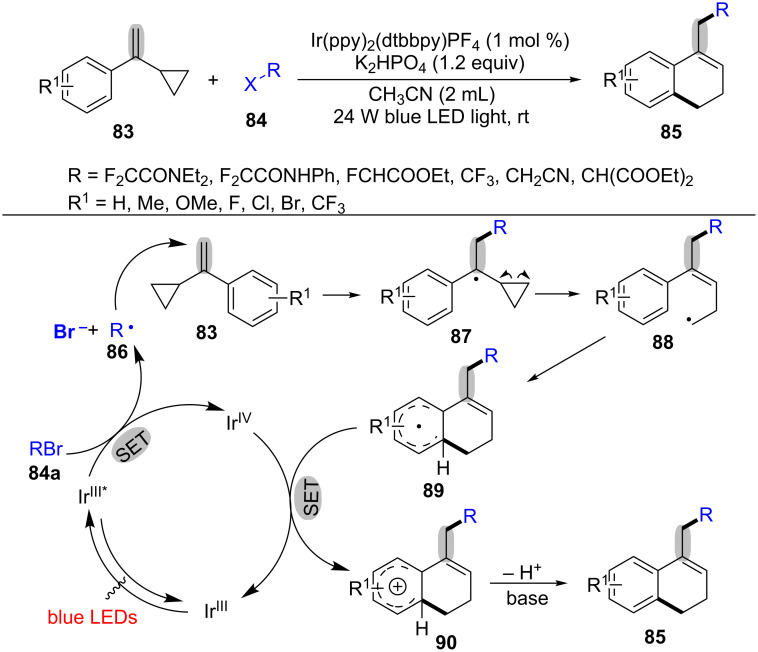
Visible-light-induced alkylation/ring-opening/cyclization of cyclopropyl olefins with bromides.

In 2016, Li’s group reported a photoredox catalysis oxidative radical ring-opening and cyclization of cyclopropyl olefins **83** with bromides **84** for the synthesis of partially saturated naphthalenes **85** in moderate to excellent yields (Scheme 19) [97]. It was the first example for alkylation, ring-opening and cyclization cascade reaction of the cyclopropyl olefins under photoredox catalysis. The alkylation reagents could be extended to other bromides, such as monofluoro-substituted bromides, trifluoro-substituted bromides, bromoacetonitrile and bromomalonate. This alkylation/ring-opening/cyclization was carried out by using Ir(ppy)_2_(dtbbpy)PF_4_ as photocatalyst, and K_2_HPO_4_ as base in MeCN under the irradiation of 24 W blue LED light at room temperature for 12–36 h. A plausible mechanism is shown in Scheme 19. Firstly, the substrate **84a** underwent oxidative quenching under the action of an iridium photoredox catalyst to afford the alkyl radical **86**, which adds to the C–C double bond of MCPs **83** to deliver the benzyl radical **87**. Then, it undergoes a ring-opening process to afford the terminal alkyl radical **88**. Next, the alkyl radical **88** intramolecular cyclizes with the phenyl ring to give intermediate **89**. Finally, the resulting aryl radical intermediate **89** is oxidized and deprotonated to provide the target product **85**. In the process, two new C–C bonds and a new ring are formed.

### Oxidative radical ring-opening of cyclopropanols

**Scheme 20:**
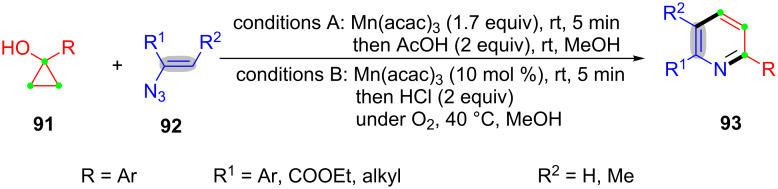
Mn(III)-mediated ring-opening and [3 + 3]-annulation of cyclopropanols and vinyl azides.

In 2011, Chiba’s group presented Mn(III)-mediated ring-opening and [3 + 3]-annulation of cyclopropanols **91** and vinyl azides **92** for the synthesis of azaheterocyles **93** (Scheme 20) [98]. This strategy could also be applied to the synthesis of the quaternary indole alkaloid and melinonine-E.

**Scheme 21:**
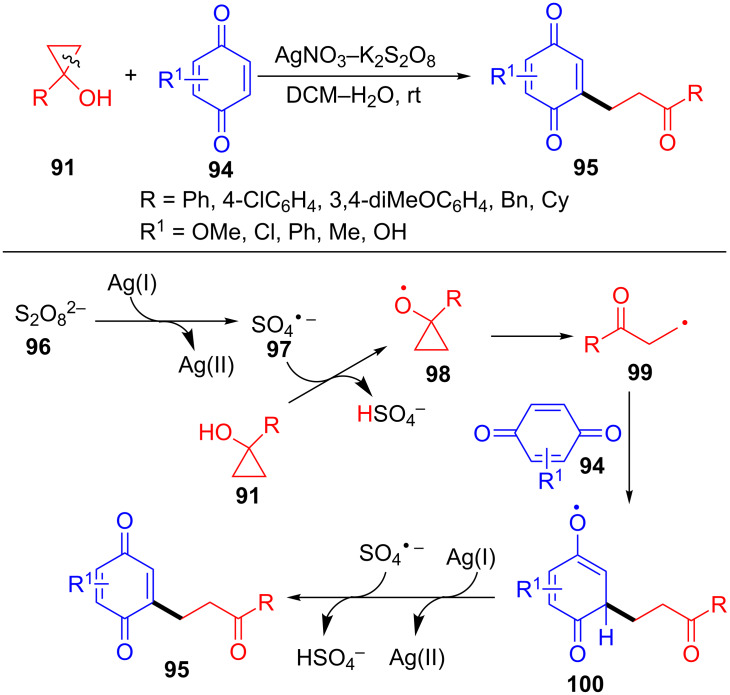
Ag(I)-catalyzed oxidative ring-opening of cyclopropanols with quinones.

Quinones play an important role in organic chemistry because of their unique structure. In 2013, Malayappasamy and co-workers reported an efficient and convenient method for the synthesis of γ-carbonyl quinones **95** via ring-opening and functionalization of cyclopropanols **91** with quinones **94** (Scheme 21) [99]. In this transformation, both AgNO_3_ and FeSO_4_ were all efficient catalysts for the ring-opening and functionalization reaction. However, AgNO_3_ was superior than FeSO_4_ according to the reaction yields and time. Interestingly, aromatic cyclopropanols delivered higher yields than aliphatic ones. The mechanism for the Ag(I)-catalyzed oxidative ring-opening and functionalization of cyclopropanols with quinones is outlined in Scheme 21. Firstly, the sulfate radical anion **97** is generated from persulfate **96** under the action of Ag(I). Next, the radical **97** reacts with cyclopropanol **91** to give the cyclopropoxy radical **98**, which undergoes a ring-opening process to produce β-keto radical **99**. The radical **100** is formed through the addition of radical **99** to the quinones **94**. Finally, the intermediate **100** occurres reoxidation with Ag(II) to provide the final product **95** along with regenerated Ag(I).

**Scheme 22:**
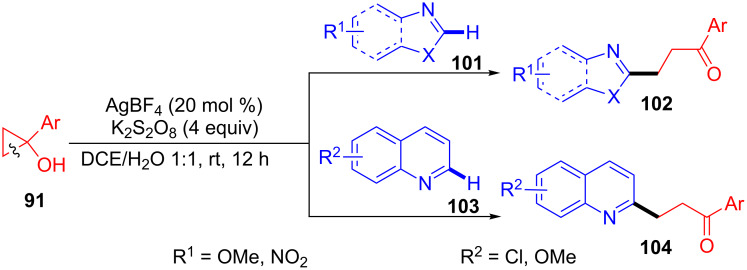
Ag(I)-catalyzed oxidative ring-opening of cyclopropanols with heteroarenes.

In 2015, Duan et al. developed a Ag(I)-catalyzed oxidative ring-opening of cyclopropanols **91** with heteroarenes **101** or **103** for the synthesis of carbonyl-containing alkyl-substituted heteroarenes **102** or **104** under mild conditions in moderate to good yields with good functional group tolerance (Scheme 22) [100]. This reaction went through a selective C(sp^3^)–C(sp^3^) bond cleavage, C–H activation and C(sp^3^)–C(sp^2^) bond formation. Notably, this finding was the first example for silver-catalyzed regioselective C2-alkylation of heterorarenes with primary alkyl radicals, generated from cyclopropanols through a radical ring-opening process.

**Scheme 23:**
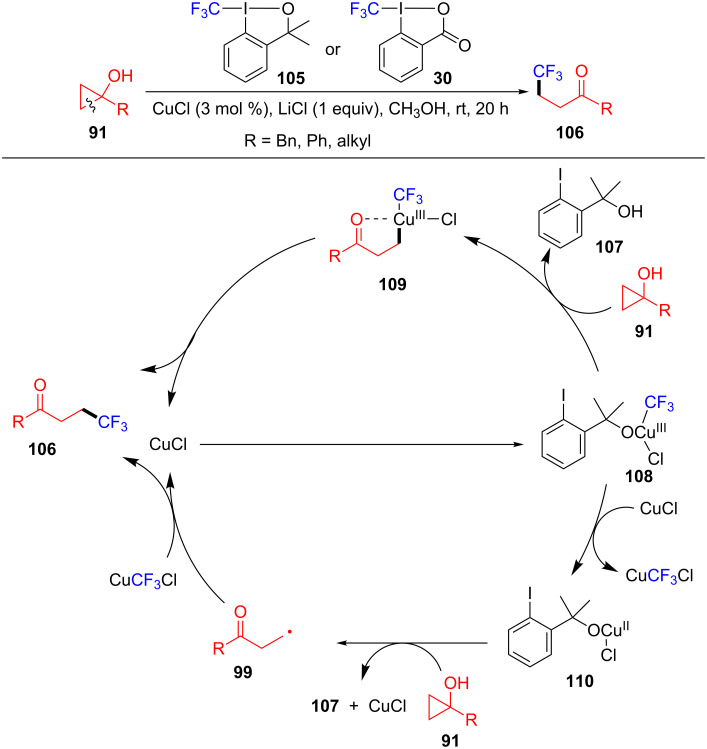
Cu(I)-catalyzed oxidative ring-opening/trifluoromethylation of cyclopropanols.

Lopp’s group also reported an efficient approach for copper-catalyzed ring-opening and trifluoromethylation of cyclopropanols **91** to construct β-trifluoromethyl-substituted ketones **106** (Scheme 23) [101]. Additionally, a series of cyclopropanols with different functional R groups were successfully scaled up to 1 mmol. In this transformation, there exist two possible pathways to produce the target product **106**. The Togni reagent (**105**) reacts with CuCl to generate Cu(III) complex **108**. Then, the intermediated **109** is generated from the electrophilic attack of copper(III) **108** with cyclopropanol **91**. Finally, the desired product **106** is formed through reductive elimination of CuCl in intermediated **109**. On the other hand, the intermediated **108** can lose the CF_3_ radical to generate the Cu(II) complex **110**. Next, the complex **110** reacts with **91** to give the radical **99**. The desired product **106** was produced by the interception of the CF_3_ radical, which came from CuCF_3_Cl.

**Scheme 24:**
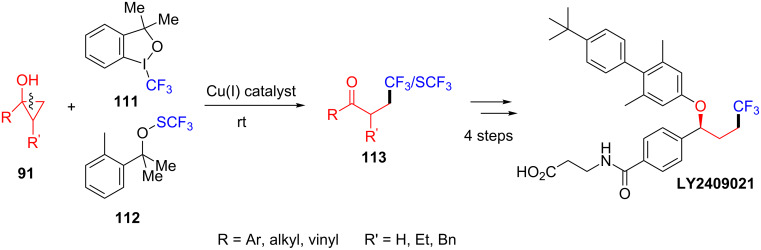
Cu(I)-catalyzed oxidative ring-opening and trifluoromethylation/trifluoromethylthiolation of cyclopropanols.

In the same year, Dai’s group also reported a copper-catalyzed ring-opening and trifluoromethylation or trifluoromethylthiolation of cyclopropanols **91** for the synthesis of β-CF_3_/SCF_3_-substituted ketones **113** (Scheme 24) [102]. This strategy was also applied to the synthesis of LY2409021. The LY2409021 was a glucagon receptor antagonist and used in clinical trials for type 2 diabetes mellitus. Xu et al. also presented the similar ring-opening/trifluoromethylation of cyclopropanols for the synthesis of various β-trifluoromethyl ketones [103].

**Scheme 25:**
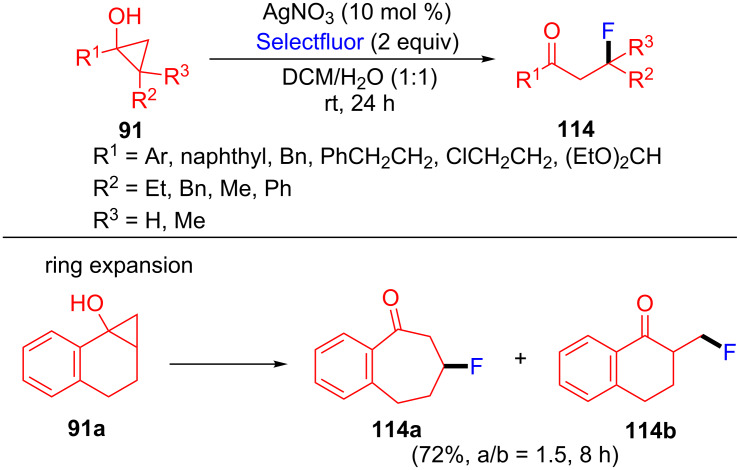
Ag(I)-mediated oxidative ring-opening/fluorination of cyclopropanols with Selectfluor.

In this year, Loh et al. [104] and Zhu et al. [105] proposed a oxidative ring-opening and fluorination of cyclopropanols **91** with Selectfluor to construct β-fluorinated ketones **114** (Scheme 25). In Loh’s work, the Fe(III)- or Ag(I)-catalyzed oxidative ring-opening and fluorination of cyclopropanols **91** via radical rearrangement is disclosed. Notably, this reaction proceeds at room temperature and tolerates a diverse array of cyclopropanols. In Zhu’s work, the fluorination of **91a** was notable because the seven-membered cyclic product **114a** and five-membered cyclic product **114b** were formed.

**Scheme 26:**
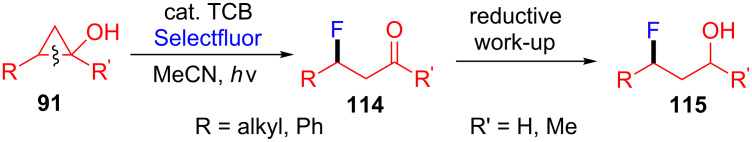
Photocatalyzed ring-opening/fluorination of cyclopropanols with Selectfluor.

Lectka’s group also presented a new approach to β-fluorinated ketones **114** via photocatalyzed ring-opening and fluorination of cyclopropanols **91** with Selectfluor under mild and simple conditions (Scheme 26) [106]. It is worth mentioning that a number of electronically and sterically diverse β-fluorinated carbonyl-containing compounds **114** and γ-fluoro alcohols **115** could be prepared through this method.

**Scheme 27:**
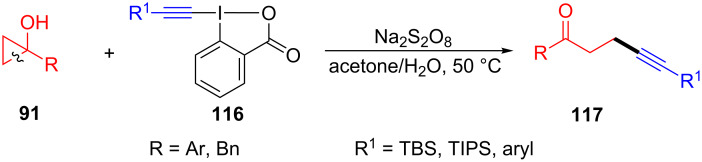
Na_2_S_2_O_8_-promoted ring-opening/alkynylation of cyclopropanols with EBX.

In 2015, Duan and co-workers introduced the Na_2_S_2_O_8_-promoted ring-opening/alkynylation of cyclopropanols **91** with ethynylbenziodoxolones (EBX) **116** for the synthesis of the alkynylated ketones **117** (Scheme 27) [107]. This reaction involved a C–C bond cleavage, radical rearrangement, and C–C bond formation, and showed a wide substrates scope under mild conditions. Surprisingly, four- and five-membered cycloalkanols were suitable in this system.

**Scheme 28:**
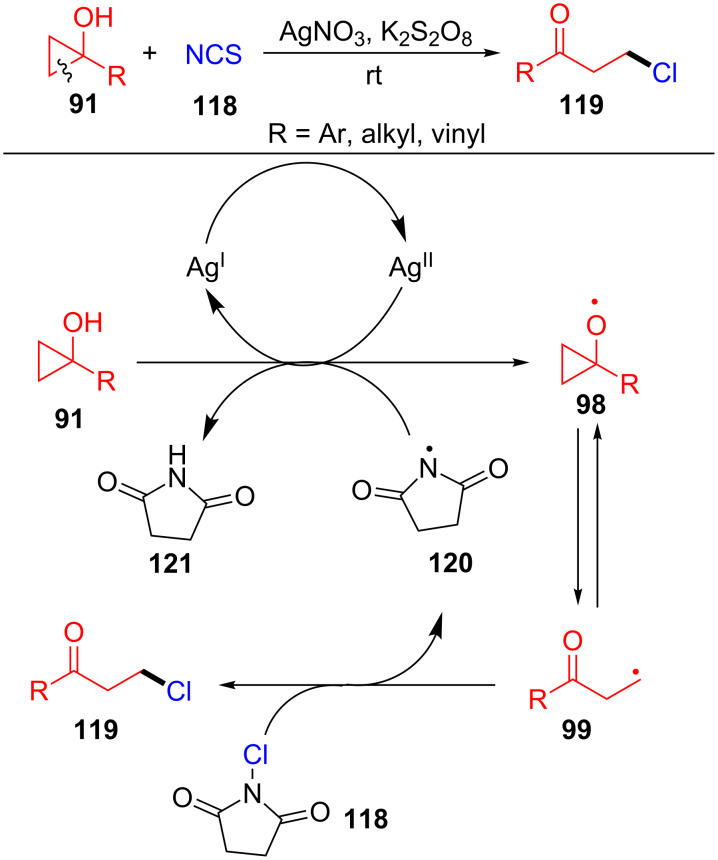
Ag(I)-catalyzed ring-opening and chlorination of cyclopropanols with aldehydes.

In 2015, Zhu’s group developed the silver-catalyzed ring-opening of cycloalkanols **91** with NCS **118** for the synthesis of distally chlorinated ketones **119** (Scheme 28) [108]. The reaction was carried out with inexpensive reagents and can also be applied to the distal bromination of cycloalkanols. The possible mechanism is outlined in Scheme 28. The cycloalkoxy radical **98** is generated from cyclopropanol **91** under the action of the metastable Ag(II) species, which is formed by the interaction of AgNO_3_ and K_2_S_2_O_8_. The radical **98** undergoes a ring-opening to give the alkyl radical **99**. Finally, the radical **99** is intercepted by NCS **118** to furnish the chlorinated ketone **119**. The generated imidyl radical **120** can also participate in hydrogen abstraction of cyclopropanol **91** to form the radical **98**.

**Scheme 29:**
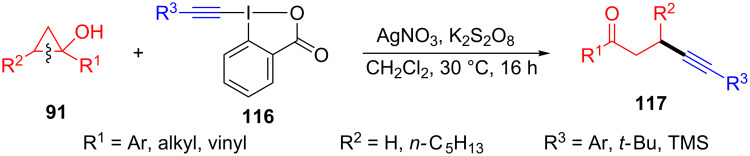
Ag(I)-catalyzed ring-opening/alkynylation of cyclopropanols with EBX.

In 2016, the silver-promoted oxidative ring-opening/alkynylation of cyclopropanols **91** with ethynylbenziodoxolones (EBX) **116** had been presented by Li and co-workers (Scheme 29) [109]. Both silver(I) nitrate and potassium persulfate played an important role in this transformation.

**Scheme 30:**
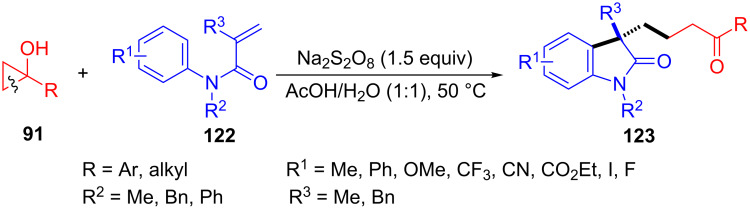
Na_2_S_2_O_8_-promoted ring-opening/alkylation of cyclopropanols with acrylamides.

In 2016, Hu and co-workers developed a novel ring-opening of cyclopropanols **91** with acrylamides **122** for the synthesis of oxindoles **123** (Scheme 30) [110]. A series of desired γ-carbonylalkyl-substituted oxindoles **123** were synthesized between *N*-phenyl acrylamides **122** and tertiary cyclopropanols **91** through Na_2_S_2_O_8_-promoted radical cyclization under transition-metal free conditions. With the addition of a radical scavenger such as TEMPO or BHT, the reaction was suppressed remarkably.

**Scheme 31:**
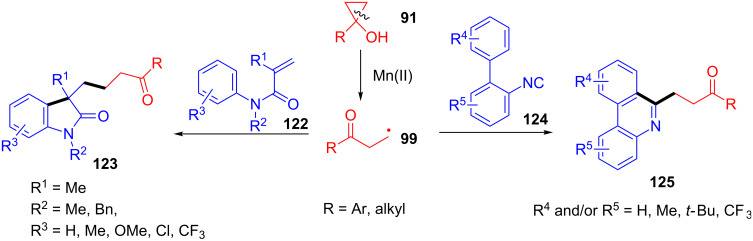
Cyclopropanol ring-opening initiated tandem cyclization with acrylamides or 2-isocyanobiphenyls.

In the same year, Dai’s group also reported the ring-opening-initiated tandem cyclization of cyclopropanols **91** with acrylamides **122** or 2-isocyanobiphenyls **124** (Scheme 31) [111]. This transformation involved a C–C bond cleavage and two C–C bond formations, and showed excellent functional group tolerance, satisfactory yields and operational simplicity.

**Scheme 32:**
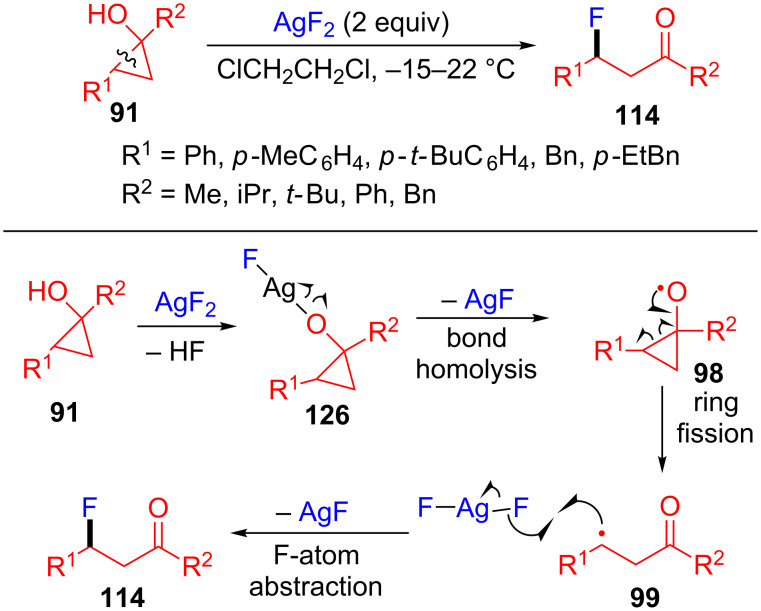
Ag(II)-mediated oxidative ring-opening/fluorination of cyclopropanols with AgF_2_.

In 2017, Mohr’s group proposed a straightforward approach to synthesize β-fluorinated ketones **114** by using AgF_2_ as both oxidant and fluorine atom source via the silver(II)-mediated ring-opening and fluorination of cyclopropanols **91** (Scheme 32) [112]. Through this method, a fluorine atom could easily be introduced in the β-position of a ketone. The mechanism is outlined in Scheme 32, the Ag-alkoxide complex **126** is initially formed from the process of ligand exchange between the substrate and AgF_2_. The alkoxy radical **98** is produced via a single-electron oxidation by Ag–O bond homolysis. As a feature of the cyclopropane system, the radical **98** goes through a ring fission to form the alkyl radical **99**. Finally, the radical **99** abstracted an F-atom from another molecule of AgF_2_ to produce the target product **114**.

**Scheme 33:**
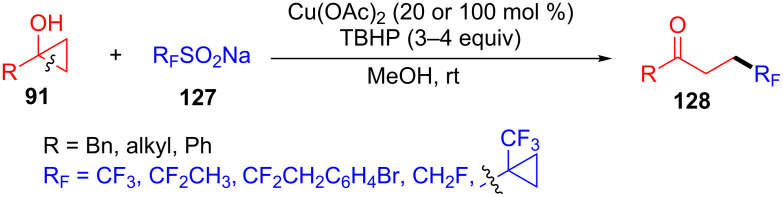
Cu(II)-catalyzed ring-opening/fluoromethylation of cyclopropanols with sulfinate salts.

Kananovich and co-workers demonstrated the copper-catalyzed ring-opening and trifluoromethylation of teriary cyclopropanols **91** with fluorinated sulfinate salts **127** for the synthesis of β-trifluoromethyl ketones **128** at room temperature and in an open flask (Scheme 33) [113]. The presented results provided an efficient and convenient method for the synthesis of diverse fulorinated ketones from cyclopropanols.

**Scheme 34:**
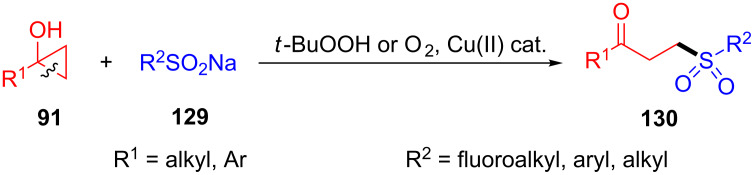
Cu(II)-catalyzed ring-opening/sulfonylation of cyclopropanols with sulfinate salts.

In the same year, this group developed a similar copper-catalyzed ring-opening and sulfonylation of teriary cyclopropanols **91** with sodium sulfinates **129** for the synthesis of γ-keto sulfones **130** in excellent yields (Scheme 34) [114]. The reaction was compatible with a series of fluoroalkyl, aryl and alkyl sulfinate salts. Notably, oxygen instead of THBP as oxidation was viable in this transformation.

**Scheme 35:**
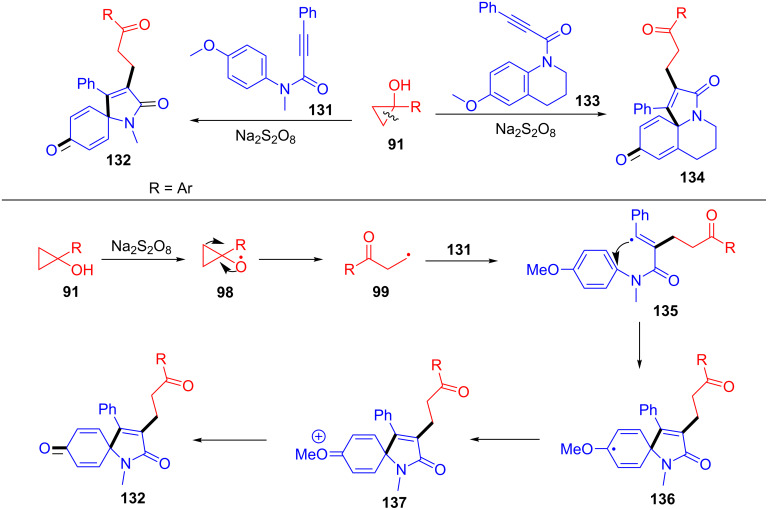
Na_2_S_2_O_8_-promoted ring-opening/arylation of cyclopropanols with propiolamides.

In 2017, Reddy and co-workers reported the first radical cyclization of propiolamides (**131** and **133**) with cyclopropanols **91** for the synthesis of azaspiro[4.5]deca-3,6,9-triene-2,8-diones **132** and 6,7-dihydro-3*H*-pyrrolo[2,1-*j*]quinoline-3,9(5*H*)-diones **134** (Scheme 35) [115]. Interestingly, this transformation proceeded under transition-metal-free conditions with high selectivity and yields. A series of substituents such as methoxy, dimethoxy, trimethoxy, methyl, chloro, bromo, and fluoro on the aromatic ring of cyclopropanols were tolerated well. The mechanism is outlined in Scheme 35. A β-carbonylalkyl radical **99** is produced from cyclopropanol **91** through a SET process. Then, addition of the radical **99** at the α-position of carbonyl in the substrate **131** furnishes the vinyl radical **135**. Next, the vinyl radical **135** occurred 5-*exo* cyclization with the phenyl ring to generate the intermediate **136**. Finally, the intermediate **136** underwent oxidation and deprotonation to give the desired product **132**.

**Scheme 36:**
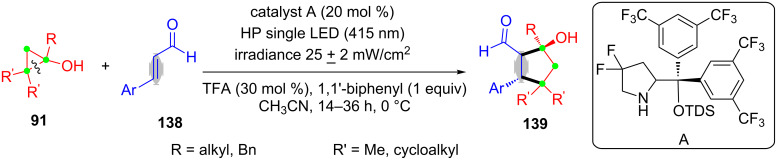
The ring-opening and [3 + 2]-annulation of cyclopropanols with α,β-unsaturated aldehydes.

In this year, Melchiorre’s group reported the ring-opening and [3 + 2]-annulation of cyclopropanols **91** with α,β-unsaturated aldehydes **138** for the synthesis of stereochemically dense cyclopentanols **139** with excellent enantioselectivity (Scheme 36) [116]. This transformation merged a stereocontrolled radical pattern with a classical ionic process in a cascade sequence.

**Scheme 37:**
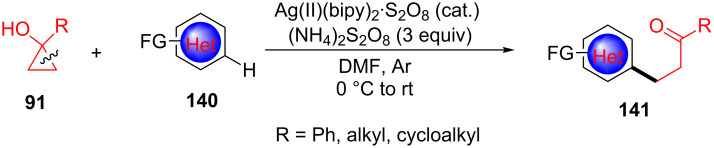
Cu(II)-catalyzed ring-opening/arylation of cyclopropanols with aromatic nitrogen heterocyles.

In 2018, Orellana et al. developed the Ag(II)-catalyzed ring-opening and functionalization of cyclopropanols **91** with electron-poor aromatic nitrogen heterocyles **140** under acid-free conditions and used a well-defined catalyst [Ag(II)(bipy)_2_S_2_O_8_] at low loadings (Scheme 37) [117]. This finding indicated that the silver pyridine complex plays an important role in single electron oxidants of cyclopropanols.

**Scheme 38:**
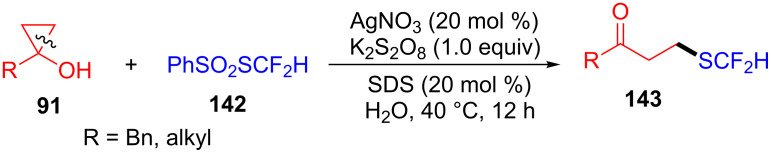
Ag(I)-catalyzed ring-opening and difluoromethylthiolation of cyclopropanols with PhSO_2_SCF_2_H.

In the same year, a silver-catalyzed ring-opening and difluoromethylthiolation of cyclopropanols **91** with PhSO_2_SCF_2_H **142** for the synthesis of difluoromethylthioethers **143** was reported by Shen and co-workers (Scheme 38) [118]. AgNO_3_ was utilized as catalyst, K_2_S_2_O_8_ as oxidant, and SDS (sodium dodecyl sulfate) as addictive in water. The SDS plays a key role in this transformation, and it enhances the solubility of both reactants in water. The cycloalkanol derivatives with electron-rich substituents on the phenyl rings deliver the corresponding products in higher yields than that with electron-deficient substituents.

**Scheme 39:**
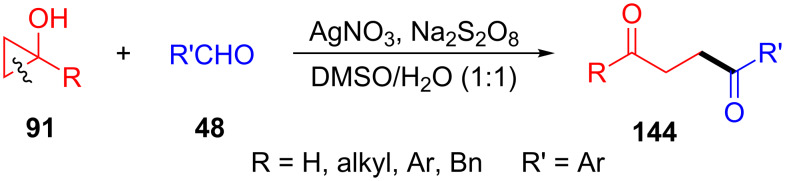
Ag(I)-catalyzed ring-opening and acylation of cyclopropanols with aldehydes.

In 2018, Zhu and co-workers also reported the first silver-catalyzed ring-opening and acylation of cyclopropanols **91** with aldehydes **48** for the synthesis of 1,4-diketones **144** (Scheme 39) [119]. They proposed that the involvement of an uncommon water-assisted 1,2-HAT process was strongly exothermic and it promoted the addition of alkyl radicals to C=O bonds in aldehydes. The electronic effect of the phenyl rings in the aldehydes showed important influence on the reaction yields.

**Scheme 40:**
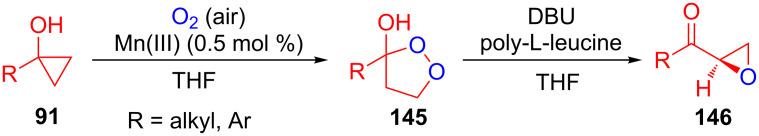
Aerobic oxidation ring-opening of cyclopropanols for the synthesis of 2-oxyranyl ketones.

In 2017, Kananovich developed a simple and efficient one-pot method for the preparation of enantiomerically enriched 2-oxyranyl ketones **146** by aerobic oxidation of easily available cyclopropanols **91** via intermediate formation of peroxyketone intermediates **145**, followed by enantioselective epoxide formation in the presence of a poly-L-leucine catalyst and DBU (Scheme 40) [120].

**Scheme 41:**
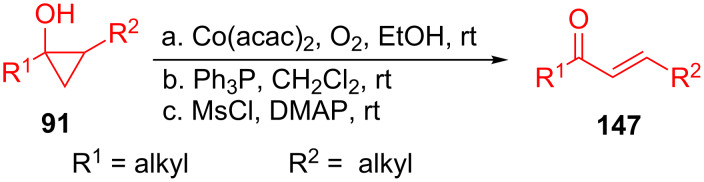
Aerobic oxidation ring-opening of cyclopropanols for the synthesis of linear enones.

In 2014, a practical method for the conversion of 1,2-disubstituted cyclopropanols **91** derived from Kulinkovich cyclopropanation into linear enones **147** was developed by Wu and co-workers [121]. The approach features the regioselective cleavage of the cyclopropane rings in EtOH at room temperature with cheap and readily available Co(acac)_2_ as the catalyst and air as the reagent (Scheme 41).

**Scheme 42:**
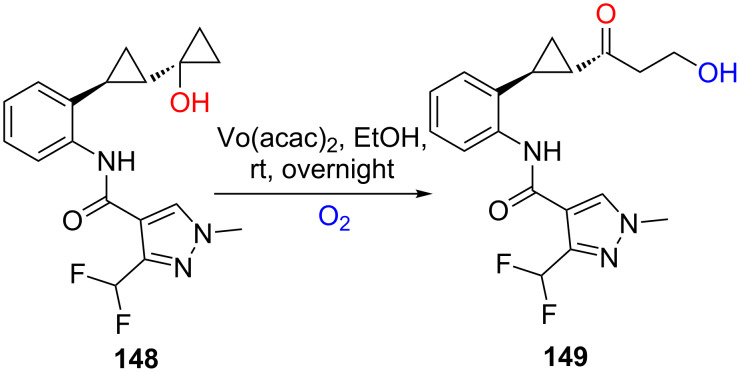
Aerobic oxidation ring-opening of cyclopropanols for the synthesis of metabolite.

In 2015, Tyagi’s group presented a biomimetic synthesis of metabolite **149** from intermediate **148** by using catalytic vanadyl acetylacetonate and molecular O_2_ (Scheme 42) [122]. The transformation went through aerobic oxidation ring-opening of cyclopropanols. The results showed that the oxygen atom of newly-formed hydroxy group came from molecular O_2_.

## Conclusion

In the past 20 years, the field of oxidative radical ring-opening/cyclization of cyclopropane derivatives (including methylenecyclopropanes, cyclopropyl olefins and cyclopropanols) has experienced significant advances. This utility has been highlighted in a number of complex natural product syntheses. In this review, we have systematically summarized various oxidative radical strategies developed for the ring-opening and cyclization of cyclopropane derivatives. Despite these advances, there still exist opportunities for exploration and many questions to be addressed. Although oxidative radical ring-opening/cyclization of functionalized cyclopropane derivatives has been well developed, the ring-opening/cyclization of common cyclopropane derivatives is conspicuously absent. On the other hand, green and environmentally friendly strategies, such as photocatalysis or electrocatalysis, can be another orientation for further developments. This review opens the scope for future developments in new methodologies which promise the synthesis of novel fused cyclic systems with a wide range of medicinal and synthetic applications.
